# Collective states and their transitions in football

**DOI:** 10.1371/journal.pone.0251970

**Published:** 2021-05-24

**Authors:** Mitchell Welch, Timothy M. Schaerf, Aron Murphy

**Affiliations:** 1 School of Science and Technology, University of New England, Armidale, New South Wales, Australia; 2 Faculty of Medicine, Nursing and Midwifery & Health, University of Notre Dame Australia, Sydney, New South Wales, Australia; Utrecht University, NETHERLANDS

## Abstract

Movement, positioning and coordination of player formations is a key aspect for the performance of teams within field-based sports. The increased availability of player tracking data has given rise to numerous studies that focus on the relationship between simple descriptive statistics surrounding team formation and performance. While these existing approaches have provided a high-level a view of team-based spatial formations, there is limited research on the nature of collective movement across players within teams and the establishment of stable collective states within game play. This study draws inspiration from the analysis of collective movement in nature, such as that observed within schools of fish and flocking birds, to explore the existence of collective states within the phases of play in soccer. Order parameters and metrics describing group motion and shape are derived from player movement tracks to uncover the nature of the team’s collective states and transitions. This represents a unique addition to the current body of work around the analysis of player movement in team sports. The results from this study demonstrate that sequences of ordered collective behaviours exist with relatively rapid transitions between highly aligned polar and un-ordered swarm behaviours (and vice-versa). Defensive phases of play have a higher proportion of ordered team movement than attacking phases, indicating that movements linked with attacking tactics, such as player dispersion to generate passing and shooting opportunities leads to lower overall collective order. Exploration within this study suggests that defensive tactics, such as reducing the depth or width to close passing opportunities, allows for higher team movement speeds and increased levels of collective order. This study provides a novel view of player movement by visualising the collective states present across the phases of play in football.

## Introduction

Collective movement is a common phenomenon that can be seen across a variety of biological systems that range in scale from cells moving within living organisms, crowds of people interacting within thoroughfares, through to herds of large mammals. This phenomena, usually referred to as “flocking” in birds, “schooling” or”shoaling” in fish, “swarming” in insects or “herd” behaviour in mammals has evolved to serve a range of functions across different species [1]. Theoretical studies have shown that relatively simple interactions between individuals, and between individuals and their environment, can give rise to complex, spectacular, self-organising, visual displays of collective movement that emerge in the absence of centralised control [2–4]. The broad hypothesis is that collective motion of animal groups arise due to application of simple rules of interaction between individuals, similar to those used in theoretical models [5]. Collective motion has proved to be a rich field of study [1], with the development of many models that incorporate individual-level interactions to simulate the group-level patterns produced by a wide range of biological systems [2–4, 6–8] and, more recently, methods for inferring the presence and form of such interactions directly from observations of animals [1, 9–18].

The collective patterns that emerge in nature commonly consist of periods with relatively ordered group movement, where individuals are well aligned and moving with consistent speeds, and disordered periods of group movement, where individuals position themselves to remain part of the group, but without alignment of movement and matching of speeds between individual. Variations of these group-level behaviours can lead to toroidal (milling) formations with high local alignment but global rotation and dynamic formations with varying densities of individuals and levels of individual alignment [4, 18]. Modelling work that simulates the interactions between individuals within a group through discrete behavioural zones has demonstrated that the group formations observed in nature represent a finite number of collective states. These states can achieve stability under the right conditions, with multiple different stable states appearing for the same individual-level behaviour. Transitions between the collective states occur when there is a perturbation that provides sufficient disruption to the alignment, position and speeds of the individuals within the group. This causes the formation to collapse into an unstable transitional scheme. Transitional periods between the identifiable collective states are relatively short and either result in the group moving from one stable state to another, or a movement back to the starting state depending on the nature and scale of the disturbance to the group [19].

The collective behaviours that have been observed within groups of individuals in nature (such as coordinated aligned movement, and expansion and contraction of the group) can also be observed amongst groups of players in team-based invasion-type field sports (for example, soccer or rugby). Several studies have demonstrated that teams participating in field sports can be viewed as dynamical systems composed of individual agents that form collective patterns, within the sets of constraints placed on the system, but without central control. It should be noted, however, that unlike shoals of fish or flocks of birds, competitive teams train and plan to maintain particular formations and apply specific group and individual level tactics dependent on context. System constraints can take the form of the rules of the games and transitions between sub-phases within play in response to competitive demands (e.g. chasing, blocking or re-forming). The dynamical systems approach to understanding team movements is based on observations from other semi-constrained systems of human movement such as crowd and pedestrian behaviour [20–22] and spacing/movement in vehicular traffic [23, 24], where self-organisation driven by local interactions can be observed within the constraints of the system.

In sports, Kijima, Yokoyama et al. [25] demonstrated a power-law relationship between the movement of the team front and position of the ball in soccer. Similarly, Passos and Araujo [26] identified evidence of self-organisation within field-invasion type sports by demonstrating that the distributions of statistics for individual attacker-defender interactions display a power-law relationship in soccer, rugby and basketball. The data presented by Passos and Araújo [26] also demonstrates that the systems exhibit *degeneracy*, whereby components can interact in different ways to produce similar outcomes. The study provides strong evidence that these processes are likely to be governed by local interactions that are constrained by a range of factors such as leadership, game rules, field boundaries and player roles. Passos, Araújo et al. [27] described these factors as first and second order constraints and proposed that within such constraints, a system of self-organisation emerges through the need to co-adapt to other individuals and continuously adjust behaviours to perform within the competitive environment created by the opposing players. This concept of self-organisation within constraints has been applied within models for team-sport simulation. Lauren, Quarrie et al. [28] proposed a collective motion-based simulation for rugby union, where players are represented by agents that begin their movement on fixed tracks (representing a plan or formation forming a first order constraint) for a period of time and are then governed by local interactions. Chacoma, Almeira et al. [29] developed an agent-based model for soccer that simulates a subset of the game, with three players (two attackers and a single defender). In this scenario, the defender attempts to intercept the attacking player in possession of the ball, while attackers advance and move the ball. The movement and positioning of the individual players is governed by simple interaction rules that are able to model empirical values for ball possession time and, length/number of passes.

While previous studies have demonstrated the existence of self-organisation and collective behaviour in team-based sport, there has been little attention given to the nature of the collective states exhibited by the groups of individuals or an understanding of the transitions between these within the different phases of play. Existing studies have investigated the collective behaviour (and its link to team tactics) in soccer through the analysis of simple group-level metrics [30–34]. Examples of these have included the *centroid*, *stretch* [30], *mean speed*, *and surface area* [31–33] of the group, calculated across positions of individual team members. These metrics capture the distribution of players, the motion of the team and level of expansion/contraction of players as the teams move [34]. Analysis of these metrics across different game phases (e.g. attacking and defending game phases, characterised by ball possession) has revealed several consistent patterns associated with the phase. For example, Clemente, Couceiro et al. [30] demonstrated that the surface area of a team increases when they transition to the attacking phase and contracts when returning to the defending phase. Other studies have demonstrated the application of spatial pattern analysis to understand the formations that are evident within the phases of play. In Perl [35], machine learning is applied to group spatial patterns of soccer team formations into clusters for analysis across matches. This study shows the spatial patterns within a game soccer can be organised into a finite number of formation classes present. The analysis of temporal patterns, usually referred to as *T-patterns*, has been applied to aspects of the football such as positioning within field zones and ball possession. These studies demonstrate stable sequences of play that are punctuated by attempts to disrupt the opposing teams equilibrium with the aim of producing goal scoring opportunities [36]. Extensions of this work have linked the existence of specific temporal patterns with performance, illustrating their effect on match outcomes [37].

The work by Narizuka and Yamazaki [38] investigated the statistics that govern the alignment of players during chase behaviour in soccer. In this research, group order parameters, including the *polarisation* and *circular variance* [39], are calculated across the angles between the movement vectors from players on opposing teams. The distributions of these parameters are analysed across the player interactions in the game and uncover periods of order and disorder within movement of players involved with chase behaviour. One of the key findings is the existence of a change-over point in the order measures that is linked to the existence of chase behaviour and longer passes of the ball.

Aside from the work of Narizuka and Yamazaki [38] focusing on chase events, there has been no comprehensive study in soccer (based on the analysis of order parameters) that investigates the existence of collective states within player movement or the transitions between these states during gameplay. The aim of this research is to build on the existing body of work by analysing the movement of players across the different phases of play to understand the patterns of ordered/disordered behaviour that exist and the nature of the transitions between them.

## Materials and methods

### Datasets and processing

This investigation was based on video and position data made available by Pettersen, Johansen et al. [40]. This dataset has seen wide use for a range of studies that cover topics including both performance analytics, image processing, computer vision and machine learning [41–43]. The data available contains video recordings and player positioning data for two professional soccer games hosted at Alfheim Stadium in Tromsø, Norway in 2013. The videos are generated from a fixed array of three cameras and includes individual camera recordings along with pre-processed stitched-panoramas that are constructed from the camera array [44]. These video sets provide a complete view of the full pitch, making event annotation convenient. The player positioning data is available for the home team (Tromsø IL) and is obtained from an XZY Sports Tracking system [45]. This system uses time-of-flight radio triangulation to provide highly accurate positioning at a rate of 20Hz using on-athlete transponders and fixed base stations within the stadium environment. The resulting dataset contains the position of each player (excluding the goal keeper), a heading, speed, total distance travelled, along with the time stamp and individual player identifier. The player position provided is relative to the field with the origin (0, 0) in the bottom left corner of the field. The combined data set provides player information for approx. 190 minutes (228,204 time steps) of gameplay data, with ten players on the field at any given time (the movement of the goal-keeper is not included in the analysis).

**Fig 1:**
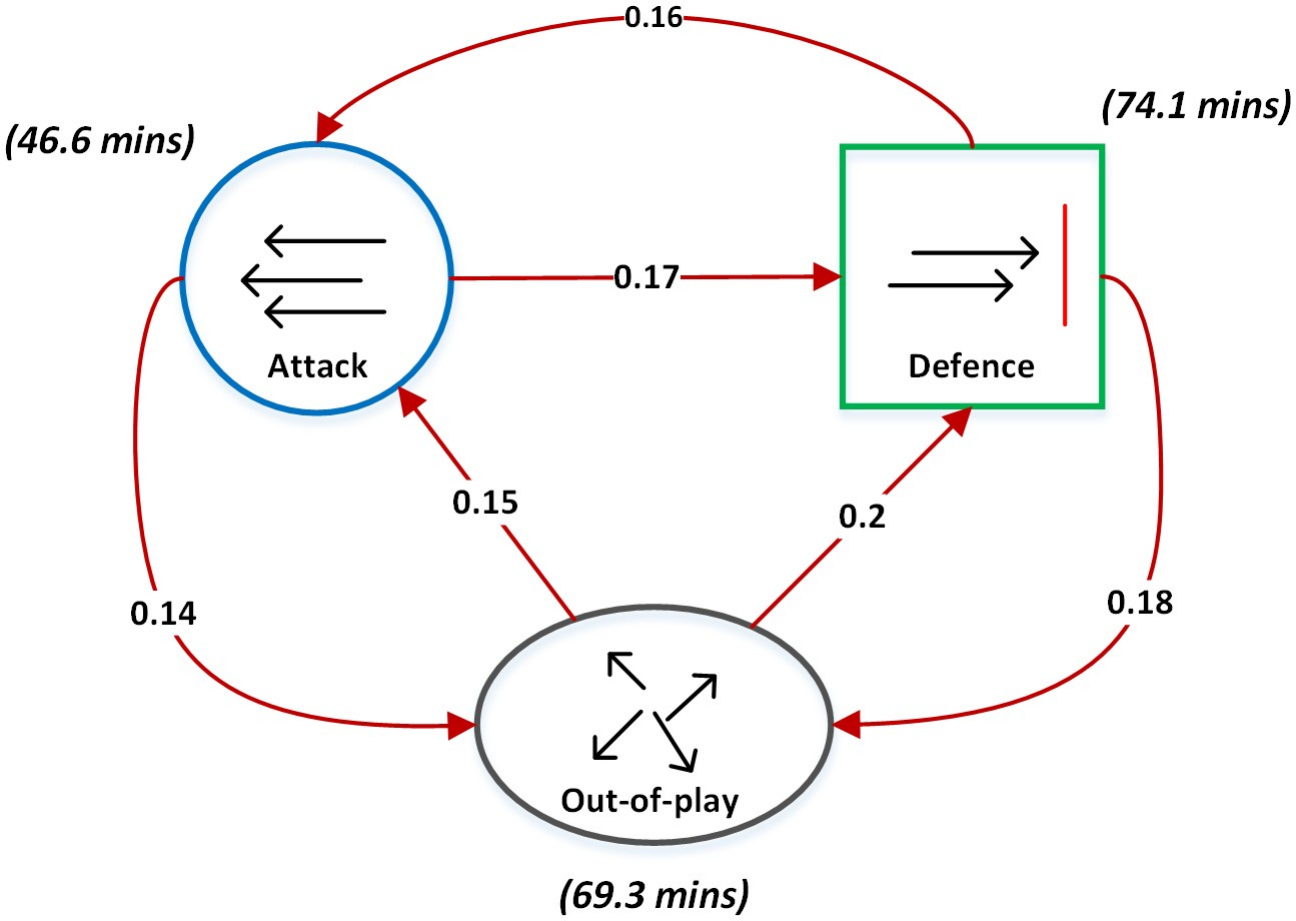
Transitions between the phases of play within games 1 and 2. The nodes represent the phase of play for the home team (blue for attack, green for defence and grey for out-of-play), the edges (in red) indicate the transition and the arrows indicate the direction of the transition. The weights on the edges represent the proportion of each transition out of all transitions that take place across the two matches analysed. The total cumulative time spent within each phase of play (in minutes) is listed with each phase.

**Fig 2:**
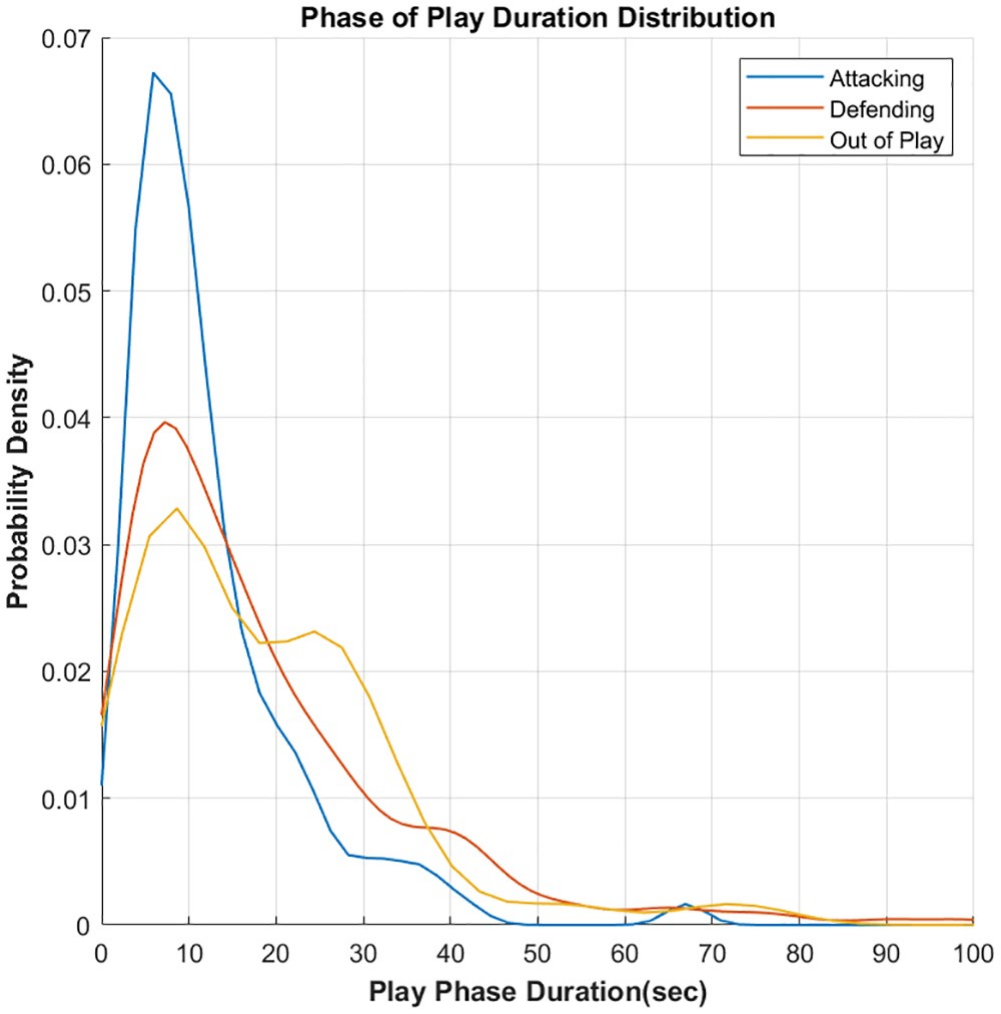
The distribution of the duration individual segments of play (i.e. ‘plays’) in each phase (Attacking, defending and out-of-play) across both games.

The video data was used to create an annotated record of the possession of the ball to infer either the *attacking* or *defending* game phase. This was achieved using the open source Visual Object Tag Tagging Tool (VoTT) [46] provided as open source software by Microsoft. The time point of each change in possession was recorded using VoTT according to the definition that is outlined by Pollard and Reep [47], where a player must have sufficient control of the ball to adjust its direction. Possession of the ball ends when the ball goes out of play, the opposing team takes possession through interception or a tackle or play is stopped for an infringement such as a foul or a player off-side. Momentary touches of the ball by players on the opposing team are not regarded as a change in possession of the ball. The video annotation data was synchronised with the player positioning data to create a complete annotated player position time-series for the home team across both games. The nature of the phase-of-play time series are summarized in Figs 1 and 2. Fig 1 provides an overview of the transitions that occur between the phases of play (along with the total time spent within each game phase). This graph consist of nodes, which represent the phases of play, and directed edges, which represent the transitions between the phases during gameplay. The weights on each edge denote the proportion of each transition as a fraction of the total number of transitions that occur across both games. Fig 2 provides an overview of the distribution of the durations of game play segments that the team spent within each game phase. These distributions were estimated using a kernel density estimator with a normally distributed kernel function.

### Order parameters and collective states

Order parameters were calculated across the player position data to describe the structure of the collective movement of the soccer team. Order parameters provide an overall measure of the order within a system and can used to describe the nature of phase changes in a physical system. Typically, they are used to describe changes between the liquid, solid, gas and plasma states of matter that take place when there is a change exerted on a physical system. Physical state changes result from interactions at the molecular level and produce the emergent transitions that are observed and characterised by order parameters calculated across the whole system. This concept has been applied to the study of self-driven individuals in biological systems, where the movement of the individuals (whether they be fish, insects etc.) is governed by decision making, environmental constraints and self-propulsion. Following on from studies that focus on collective structure in natural groups [4, 9, 18], two order parameters were calculated across the positions of all players at each time step–*polarisation* and *angular momentum*. Polarisation (*p*_*group*_), Eq (1), describes how well aligned the individuals in the group are at each time point.

$$p_{group}(t)=\frac{1}{N}|\sum_{i=1}^{N}v_{i}(t)|,$$ (1)

where ***v***_*i*_(*t*) is the unit vector in the direction of motion of player *i* at time *t* and *N* is the total number of players on the field. Values of *p*_*group*_ close to 1 indicate a high degree of alignment in the motion of players at a given time, whereas values of *p*_*group*_ closer to zero indicate a greater degree of scatter in the directions of motion.

Angular momentum (*m*_*group*_) measures the degree to which there is a consistent sense of rotation by group members about the group centre (Eq (2)).

$$m_{group}(t)=\frac{1}{N}|\sum_{i=1}^{N}{\hat{r}}_{ic}(t){\times v}_{i}(t)|,$$ (2)

where

$$r_{ic}(t)=c_{i}(t)-c_{group}(t)$$ (3)

is the vector pointing from the group centre, ***c***_*group*_(*t*) at time *t*, to the position of player *i*, ***c***_*i*_(*t*) at the same time,

$${\hat{r}}_{ic}(t)=r_{ic}(t)/|r_{ic}(t)|$$ (4)

is the corresponding unit vector, the group centre is estimated via

$$c_{group}(t)=\frac{1}{N}\sum_{i=1}^{N}c_{i}(t),$$ (5)

and *N* is the total number of players on the field at time *t*. Values of *m*_*group*_ close to 1 indicate that team members are largely consistent in moving clockwise, or anticlockwise, about the group centre at a given time.

The time series of the two order parameters defined above allows for broad classification of patterns of team movement, with the goal of identifying and understanding the group states and transitions between them as game play proceeds. The scheme for classifying the collective states using the combination of *p*_*group*_ and *m*_*group*_ parameters is adopted from [18] and is motivated by the densities observed (in *p*_*group*_ and *m*_*group*_ space*)* for clearly identifiable formations emerging in schools of fish. Under this scheme (*p*_*group*_ > 0.65) ⋀ (*m*_*group*_ < 0.35) designates the *polar* state, (*p*_*group*_ < 0.35) ⋀ (*m*_*group*_ < 0.35) designates the *swarm* state and (*p*_*group*_ < 0.35) ⋀ (*m*_*group*_ > 0.65) designates the *milling* state. The corresponding regions are marked on the corresponding density plots using red broken lines. Where the paired values of *p*_*group*_ and *m*_*group*_ lie outside the regions defining polar, swarming or milling states, the emergent behaviour of the group is classified as transitional, where the group is between states. Under this arrangement, the group can transition between any combinations of the collective states (this includes transitioning from a collective state into the transitional scheme, and back to the original state). When the team is within a transitional phase, it has features of both ordered and unordered group structures, with combinations of polar, oscillating, wandering, or swarming formations [19]. In order to understand the relationship between the motion of the team and its collective states, the mean speed of the team’s movement is calculated across all players on the field at each time step. The mean group speed (${\overset{-}{\upsilon }}_{group}$) is calculated according Eq (6).

$${\overset{-}{\upsilon }}_{group}(t)=\frac{\left|c_{group}(t)-c_{group}(t-\Delta t)\right|}{\Delta t}$$ (6)

where Δ*t* is the constant duration between discrete data time steps. This calculation gives the speed of the centroid (*c*_*group*_) over time. A Gaussian-weighted moving average filter with a window of two seconds (40 samples) was applied to the time series data for the *p*_*group*_, *m*_*group*_ and ${\overset{-}{\upsilon }}_{group}$ parameters to remove the effects of noise introduced into the movement data from jitters in the underlying player positioning data.

**Fig 3:**
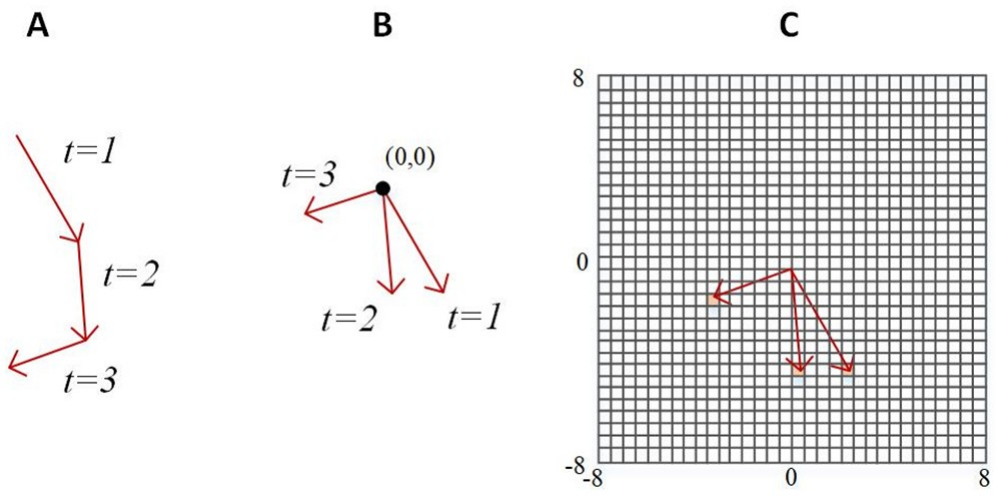
Demonstration of the steps for binning the player movement vectors for the calculation of the collective entropy *H*_*group*_. Starting with the series of movement vectors for each player for each time step within the window (in this example 3), depicted in (A), the movement vectors are transformed so that each vector starts at the origin (0,0), depicted in (B). These transformed vectors are designated to a 2-dimensional bin, depicted in (C), allowing for the calculation of P(x_ij_) for all bins.

To provide an indication of the predictability and diversity within the motion of the individual players we calculate the *Shannon Entropy*, *H*_*group*_, associated with the sequences of two-dimensional changes in position of each player within 2 second windows. For each sequence of position changes, the tail of each vector associated with a change in position is placed at the origin, (0, 0). The square domain where -8 ≤ *x* ≤ 8, -8 ≤ *y* ≤ 8 (metres) is then divided into a set of square bins of side lengths 0.5 metres, indexed via row *j*, column *i*, and resulting in a two-dimensional grid with 1024 bins. This configuration provides both sufficient resolution and range to account for the maximum individual stride lengths and sprint speeds identified by [48] over the 2 second window. For a given time window, we tally the number of times that position change vectors point into each of the bins, denoting this tally *f*_*ij*_, and aggregating data from all players into the same set of bins (a total of 400 values are used for each time window). From *f*_*ij*_, we then estimate the probability of a change in position pointing to the bin in row *j*, column *i*, via $P_{ij}=f_{ij}/\sum_{i}\sum_{j}f_{ij}$. This scheme is summarised in Fig 3. The entropy, *H*_*group*_, is then given by Eq (7).

$$H_{group}(t)=-\sum_{i=1}^{32}\sum_{j=i}^{32}P_{ij}{\text{log}}_{2}P_{ij}$$ (7)

Under this scheme, there is a maximum of 8.64 bits of information at each time step, with the 40 time step window, corresponding to two seconds of data (and the resulting 400 vectors), dictating this maximum. The aim of this overall approach is to provide an indication of the predictability of the direction and magnitude of the motion of individuals within the team at each time window. The fixed grid arrangement used for the entropy measure provides additional information on top of polarisation and angular momentum but taking to account the speed of individuals, not just angular differences between players. This provides a holistic indication of movement predictability across each time window.

Following on from previous research that examines spatial formations and collective behaviour in soccer [30, 34], the surface area *A*_*group*_(*t*) occupied by the team is calculated at each time step *t* by determining the convex hull across all player positions at time *t* and calculating the area of the resulting polygon. This was achieved by using built-in functionality within Matlab 2019a [49].

### Plots and visualisation

**Fig 4:**
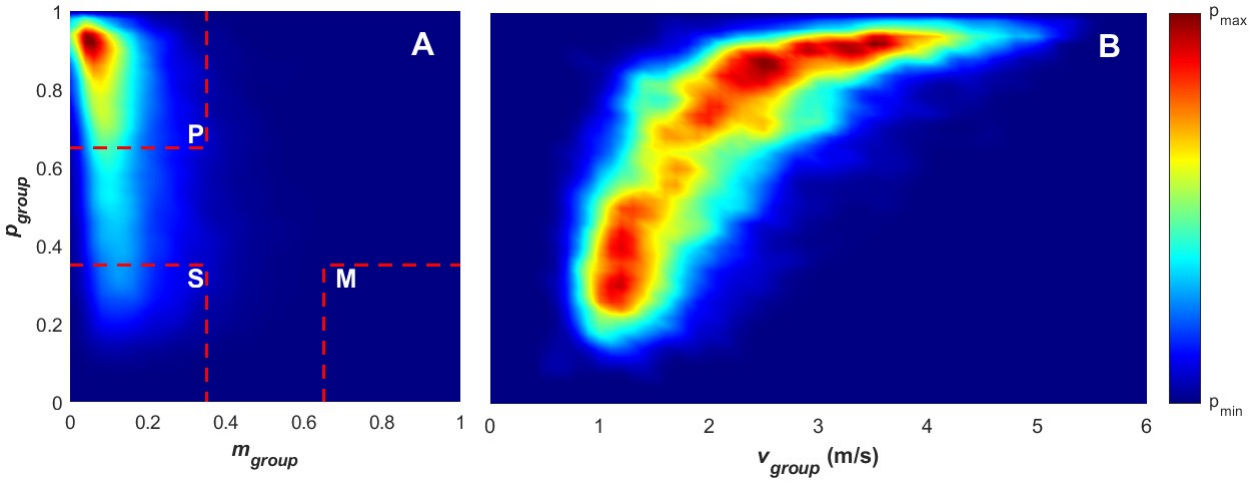
Density plots for polarisation, angular momentum and mean group speed. (A) Density plot for paired angular momentum (*m*_*group*_) and polarisation (*p*_*group*_) values, with the divisions of the order parameter space overlayed in red, indicating the polar (p), swarm (s), milling (m) and transitional states. (B) Density plot for paired mean group speed (${\overset{-}{\upsilon }}_{group}$) and polarisation (*p*_*group*_) values.

**Fig 5:**
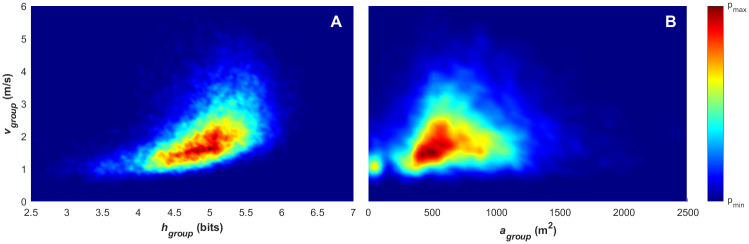
(A) Density plot for paired group entropy (*H*_*group*_) and mean group speed (${\overset{-}{\upsilon }}_{group}$) values and (B) paired surface area (*A*_*group*_) and mean group speed (${\overset{-}{\upsilon }}_{group}$) values.

**Fig 6:**
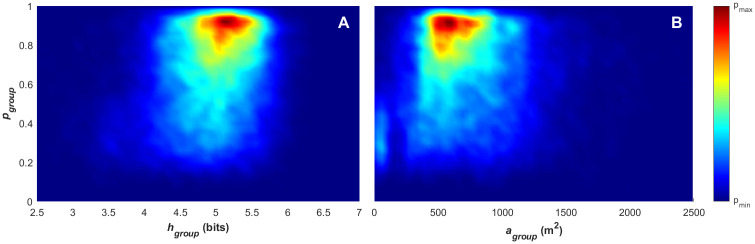
(A) Density plot for paired values of group entropy (*H*_*group*_) and polarisation (*p*_*group*_), and (B) surface area (*A*_*group*_) and mean group speed (${\overset{-}{\upsilon }}_{group}$) as.

In order to analyse and understand the collective states, their transitions and relationship with the phases of play, the order parameters were visualised through the use of probability density plots. This approach has been widely employed in previous research, both on coordinate-space data to describe relative position and motion of group-mates under different conditions [14, 17] and on order parameter-space data to describe the nature of collective states and their transitions [18]. The approach chosen for this analysis mirrors that used by Tunstrøm, Katz et al. [18], with order parameter space divided up into 50 × 50 equally spaced bins and plotting the number of order parameter-space points that fall into each using a heat map. For Figs 4B, 5 and 6, which plot ${\overset{-}{\upsilon }}_{group}$, *H*_*group*_ and *A*_*group*_ as a function of *p*_*group*_, 60 bins are used for the ${\overset{-}{\upsilon }}_{group}/A_{group}$ dimensions and 140 for the *H*_*group*_ dimension to reflect the resolution of the underlying variables. The value that is actually plotted for each bin is calculated by taking the mean across a moving window made up of the count in the target bin, along with the counts in all adjacent neighbouring bins. This has the effect of smoothing the plots. Fig 11 plots the data for time series segments that encompass the transition from one collective state (e.g. polar or swarm) to the next. These data segments were extracted by starting at the first time point in the specified starting state and ending at the last time point present in the ending state. The result is a set of time series segments for each relevant state transition that encompasses the collective state-space data in the start and end states, along with the progression through the transitional state. Quivers are overlayed on top of these density plots to denote the mean direction of movement of the group in the state space between each bin. This mean direction is calculated based upon the next value in the time series of the *p*_*group*_—*m*_*group*_ space for data each point that falls within each bin. The final component of the analysis provides a series of plots that include the player’s individual positional traces along with corresponding order parameter time-series for an exemplar transition from the polar to swarm state.

### Statistical analysis

We determined the durations spent in each observed form of collective state (swarm, polar, or transitional) during each phase of play (attacking, defending, or out-of-play). We constructed Kaplan-Meier estimates for the survival functions describing the durations spent in each collective state (Fig 8). Survival curves within each game phase were analysed for significant differences via log-rank tests [50].

## Results and discussion

### Global analysis

The summary presented in Figs 1 and 2 provides an overview of the distribution of game phases across the two games played by the monitored team. In Fig 1, we can see that the total time spent within each phase of play is skewed, with the team spending more time in the defending and out-of-play game phases. The team under study was attacking (in possession) approximately 39% of the in-play-time. This value lies within the ranges observed in other studies and is reflective of a team that is facing a higher performing opponent [51, 52]. The proportion of the game phase transitions (denoted by the red arcs) is relatively uniform, with a slight bias such that transitions into the attacking phase are less frequent, agreeing with the observation that a lower overall amount of time is spent in the attacking game phase. The distribution of durations for the individual segments of play (outlined in Fig 2) in each game phase agree with this overall trend, with a higher proportion of shorter duration segments evident in the attacking game phase, when compared to the distributions for the defending and out-of-play phases. The peaks of all three distributions lie at approximately 5–10 seconds, agreeing with distributions observed in other studies that focus on performance related to ball possession [53].

Fig 4A provides a global view of the collective states that are exhibited across the whole data set. It is immediately evident from this plot that the team spends most of its time within the polar, transitional and swarm states. The group never adopts a milling structure akin to the milling seen in fish shoals [18] (the dark-blue areas indicate areas of the state-space that are never reached). The largest proportion of time is spent in the polar state, indicating a relatively high level of alignment within the group movement across the games. The lack of time spent in the milling state is not unexpected as there are few situations within gameplay that should result in a formation that rotates about its centroid, and the linear nature of the game (e.g. moving the ball between ends of the pitch) will contribute to the polar movement pattern.

Fig 4B provides further insight, demonstrating that the polar, ordered, collective state is present primarily at higher average group speeds. This pattern could indicate that higher group speeds can only be achieved when the team’s movement is relatively ordered. This can be seen in the lack of mean team speed above 3m/s with a polarisation of less than 0.7. The apparent correlation between group speed and polarisation is consistent with coordination among individuals within the team to maintain formations when moving at higher speed, and has also been observed in animal groups, such as shoaling fish [54]. The polarised movement state likely arises from the need to coordinate spacing and trajectories so that team movement at higher speeds is made possible to meet competitive demands within the game (e.g. repositioning to reduce goal scoring opportunities while defending, creating passing opportunities while breaking through). The phenomena has been demonstrated in a human context within studies that focus on pedestrian dynamics, where microscopic movement rules tend to result in ‘lane-forming’ collective behaviour [6]. Modelling this phenomena demonstrated that the number of lanes formed within a pedestrian group scales linearly with the width of the walkway. This behaviour allows pedestrians to maximise their desired speed by avoiding costly collision. Teknomo [55] demonstrated that the average speed of the individuals decreases linearly as the density of pedestrians increases where they are moving the same direction and decreases exponentially when two-way pedestrian traffic is introduced. This two-way scenario is analogous to a formation with low polarisation and, while the environment is different and movement speeds of the individuals are on average slower (ranging between 1-2m/sec) [55], the results provide a human precedent for the effect of collective order on the movement speed of individuals.

Figs 5 and 6 provide further insight into the relationship between the mean group speed and the collective states. In Fig 5A, the density plot for mean group speed as a function of group entropy is presented. This provides a measure of the predictability of the movement of individuals within the team at each time step, with a lower group entropy (*H*_*group*_) indicating more predictable player movement within the group. Recall from the previous section that the entropy calculation is carried out across the velocity vector, so both the direction of movement and the magnitude of the movement contribute to the predictability of the player’s position as a result of their motion. Fig 5A demonstrates a positive, but non-linear, relationship between the mean group speed and entropy, with higher group speed associated with higher entropy. This indicates that when the team is moving at a higher speed, the movement of individual players is less predictable. When we compare this to Fig 6A (which provides a density plot for polarisation as a function of entropy), we can see that low entropy (< 3.5 bits) is only observed with low polarisation (< 0.5). This demonstrates that the mean speed of the player formation (i.e. the distance that the players travel) is the key driver for higher entropy. When the movement of the team is well aligned, it achieves higher mean speed (Fig 4B) and while at higher mean speeds, there is a tendency for the team to have a higher entropy (Fig 5A). The polarisation (i.e. direction of individual player movements) is a smaller contributing factor to the overall predictability of the individual movements. This demonstrates the effect on positional predictability that small angular differences have on groups of players moving at higher speeds (i.e. small differences in player alignment result in a larger dispersion of players at higher speeds, giving a higher entropy).

The team surface area as a function of mean group speed in Fig 5B and polarisation in Fig 6B, appears to be representative of multiple movement patterns. The highest group surface area is achieved at lower mean group speeds, between 1–2 m/s, with highest mean group speeds between 4–5 m/s being characteristic of mid-range surface areas (500-1000m^2^). The relationship between polarisation and surface area is relatively uniform, indicating that (at a global level) the alignment and order within the team movement is not strongly linked with its surface area.

### Collective movement analysis by game phase

**Fig 7:**
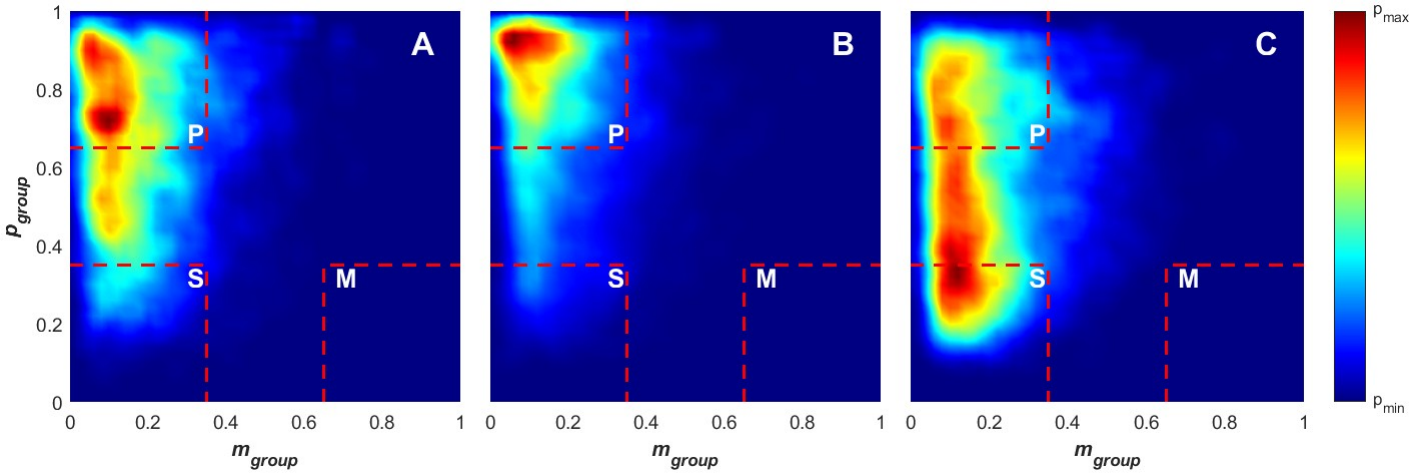
Density plots for paired angular momentum and polarisation across the attacking (A), defending (B) and out-of-play (C) game phases from both games. The divisions of the order parameter space overlayed in red, indicate the polar (p), swarm (s), milling (m) and transitional states.

This section further breaks the analysis down using the annotations for the game phases (attack, defence and out-of-play) to classify the order parameter data points for each time step. Fig 7 provides density plots for paired values of angular momentum and polarisation for the attacking (A), defending (B) and out-of-play (C) phases. This break-down demonstrates that the defending game phase (Fig 7B) has the highest degree of collective order, with the highest density area distributed mainly within the polarised state (*p*_group_ > 0.65). The density is relatively low across the transitional and swarm states. The attacking game phase (Fig 7A) demonstrates a wider distribution across the polar and transitional states (more characteristic of the global density pattern presented in Fig 4A), with the highest density still lying within the polar state. The out-of-play game phase presents the most uniform distribution across the entire range of the collective state space present in the data, with the highest density achieved in the swarm collective state. These observations agree with conventional tactics that are demonstrated within the attacking and defending phases. In a typical attacking phase, players move to create passing opportunities and counter defending players [56, 57]. This is referred to as *offensive coverage* [56] or simply *width/depth* [58] and the aim of this approach is to generate gaps within the defensive formation or isolate defenders to create 1v1 scenarios that facilitate break-throughs. The state-space distribution presented in 7A indicates that this player movement from this action results in a lower level of collective order (e.g. higher proportion of transitional and swarm state points) when compared to the defensive distribution. In a typical defending phase, players will attempt to close around the attacking players and gain numeric superiority at the point of attack (concepts of *balance*, *concentration* and *delay* [56, 58]). The state-space distribution presented in 7B indicates that this results in more ordered group movement.

**Fig 8:**
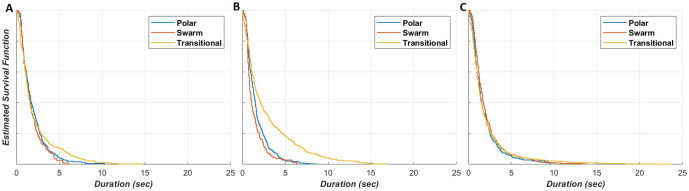
Survival curves for the durations spent in the polar, swarm and transitional collective states in the (A) attacking, (B) defending and (C) out-of-play game phases. These plots provide insight into the collective state structure within each game phase.

The survival analysis presented in Fig 8 provides insight into the durations spent within each of the collective states (polarised, swarm and transitional) across each of the game phases. Pairwise Log-rank tests across the survival functions within each game phase show that the differences in the survival curves within the attacking (8A) and out-of-play (8C) games phases were not statistically significant. In the defending phase (8B), all survival curves were statistically different: polar vs swarm (***P*** ≈ 0, DF = 1, ***χ***^**2**^ = 2.86), polar vs transitional (***P*** ≈ 0, DF = 1, ***χ***^**2**^ = 7.57) and swarm vs transitional (***P*** ≈ 0, DF = 1, ***χ***^**2**^ = 7.53). Visual inspection of the survival curves shows that in both the attacking and defending phases, instances in the transitional state tend to last longer than those spent swarming or polarised (but such differences are not statistically significant to durations of swarming and polarised motion during the attacking phase). In the defending game phase, instances in the polar state tended to be of longer duration than those in the swarm state. Within the out-of-play phase, there are no clearly observable differences between the survival curves. This analysis demonstrates that while the polar collective state is dominant (in terms of the total amount of time) in the attacking and defending phases, there are relatively long, contiguous periods where the team exhibits elements of ordered and un-ordered collective behaviour within team movement. It is evident that transitional state durations in the defensive game phase tend to be longer than in the attacking game phase (compared to the respective durations spent within the other collective states). This is likely a result of the higher proportion of time spent in the polar state during defensive phase and indicates that the polar formations are linked to longer transitions, taking more time to form and collapse in response to game play.

**Fig 9:**
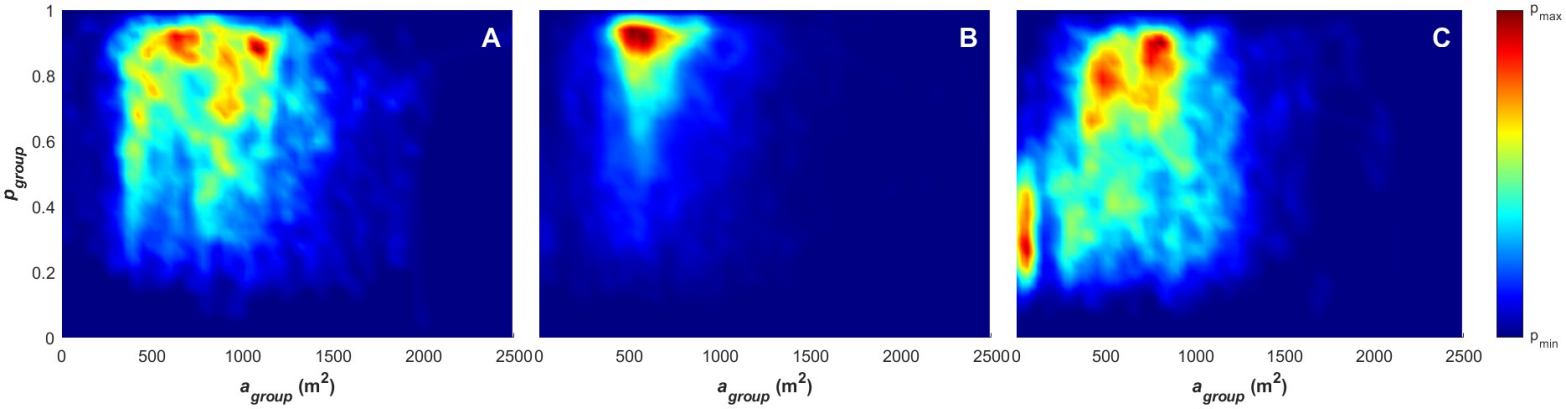
Density plots for paired group area (*A*_*group*_) and polarisation (*p*_*group*_) values across the attacking (A), defending (B) and out-of-play (C) game phases.

**Fig 10:**
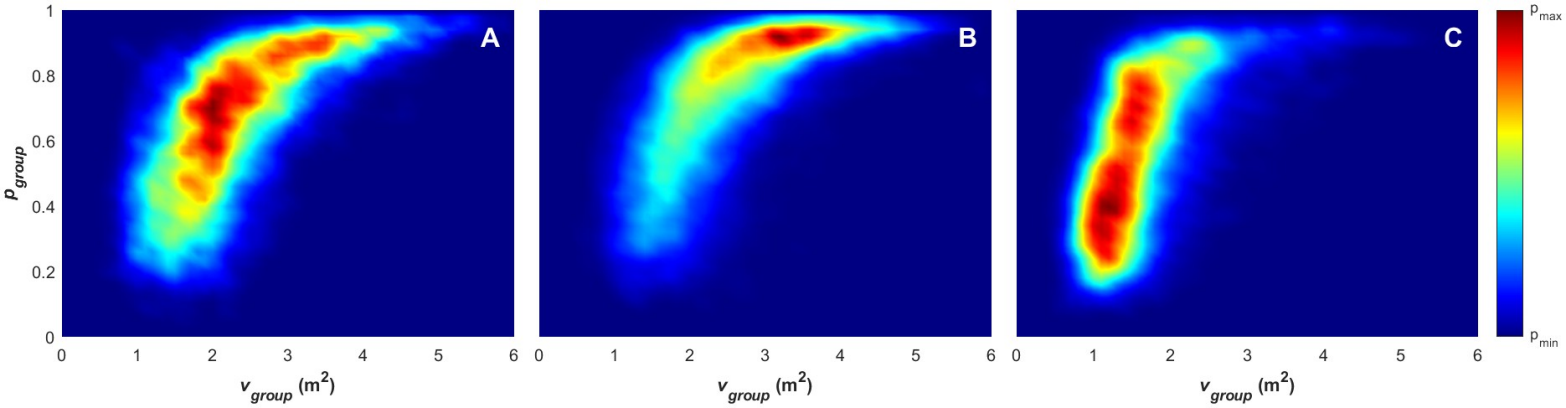
Density plots for paired mean group speed (${\overset{-}{\upsilon }}_{group}$) and polarisation (*p*_*group*_) values across the attacking (A), defending (B) and out-of-play (C) game phases.

The differences between the collective states within the defensive and attacking movement patterns are further evident within the data presented in Figs 9 and 10. Fig 9 presents the density plot for paired values of surface area and polarisation, and Fig 10 presents the density plot for paired values of mean group speed and polarisation for the attacking (9/10A), defending (9/10B) and out-of-play (9/10C) game phases respectively. The distribution of polarisation-surface area space (Fig 7) shows a large difference between the attacking and defensive phases of play. While in the attacking phase (9A), the team surface area is relatively evenly distributed across the range of 300-2000m^2^ for the polarisation range of 0.2–1. While in the defensive phase (9B), the surface area is concentrated in the range of 300-1000m^2^, for a polarisation range of 0.5–1.0. The relationship between the phase of play and the surface area of the team has been studied in a range of contexts and it has been consistently shown that defensive formations occupy a smaller surface area than attacking formations [30, 31, 59, 60]. Just as with the collective phase observations in Fig 7, this pattern of surface areas reflects conventional tactics, where attacking play leads to the team spreading out to create attacking opportunities and defensive play leads to contraction in an effort to gain numeric superiority at the point of attack.

Fig 10 presents the phase-of-play breakdown for paired values of mean group speed and polarisation in the attacking phase (10A), defending phase (10B) and out-of-play (10C). Fig 10A–10C all demonstrate the same range of mean group speeds presented in Fig 4B, however the distribution of varies greatly across each phase of play. The distribution of polarisation-mean group speed within the attacking phase is reflective of that presented in Fig 4B, with a relatively even distribution with mean group speed increasing as polarisation increases. In the defensive phase (10B), the distribution is more concentrated in the polarised state with a higher mean group speed (the area of highest concentration lies between 3-4m/s). In the out-of-play phase, the distribution is concentrated at lower mean group speeds (< 2m/s), with polarisation ranging across 0.1–0.9.

Through the game-phase breakdown in Figs 7, 9 and 10, it is evident that the defence phase consists of formations that are primarily polar, with a high level of collective order and concentrated within a relatively low surface area. The concentrated, ordered formations move with higher mean group speed. This behaviour is reflective of conventional defensive tactics, with the higher mean group speed representing more rapid movement of the concentrated formation to counter offensive play. This is in contrast to the attacking phase of the game, where there is a lower proportion of ordered collective movement, and a larger proportion of time of higher surface area formations moving with a wider distribution of mean group speeds. This is reflective of attacking manoeuvres as individual players move to occupy a larger area to create passing opportunities and gaps when facing a concentrated defence. The result of this is a collective movement pattern that has a higher proportion of transitional collective-state behaviour (with ordered and unordered components) and unordered swarm behaviour. The observation agrees with previous studies that have identified temporal patterns where attack players attempt to create space [37] and disrupt the equilibrium of the defence [36].

Movement patterns from the out-of-play phase reflect largely swarming and transitional collective-state behaviour, all with a lower mean group speed. This is reflective of a collapse in playing formations (whether they be attacking or defending) as the pressure of competitive play is removed from the game.

An interesting parallel to these patterns of collective behaviour can be seen in nature through the analysis of collective order in instances where a predator attacks individuals in a coherent moving group. There are numerous studies that use both data and simulation to investigate these relationships and evidence suggests that the risk of predation is lower when prey form ordered compact formations [15]. While specific mechanisms are not fully understood, this has been attributed to factors such as increased information transfer within ordered formations [12] and confusion of predators [61]. In this study, it can be observed that more ordered, compact and higher-speed collective movement of the defensive formation reflect patterns associated with reduced risk of predation on prey in predator-prey studies. If we draw an analogy between the defensive formations observed here in the context of football, and those associated with reduced risk of predation, then in a sense the attacking team can be thought of as ‘preying’ on the gaps that are generated by fragmenting the opposing defence.

It could then be hypothesised that the competitive demands of play lead to the evolution of the defensive tactics that employ this pattern of collective movement to reduce the attacking teams ability to successfully break through. Studies of skill development across different age groups have demonstrated that field tactics related to coordination and field usage are built up though experience and response to this competitive pressure [59, 60]. While specific situations and underpinning mechanisms for perception and decision making likely differ, it is possible that the basic drivers cited in the predator-prey examples related to cognition, confusion and information transfer may apply to the field sport scenario. This is a key point for further investigation that my provide insight into the performance of specific combinations of offensive and defensive tactics related to collective order.

### Collective state transitions

**Fig 11:**
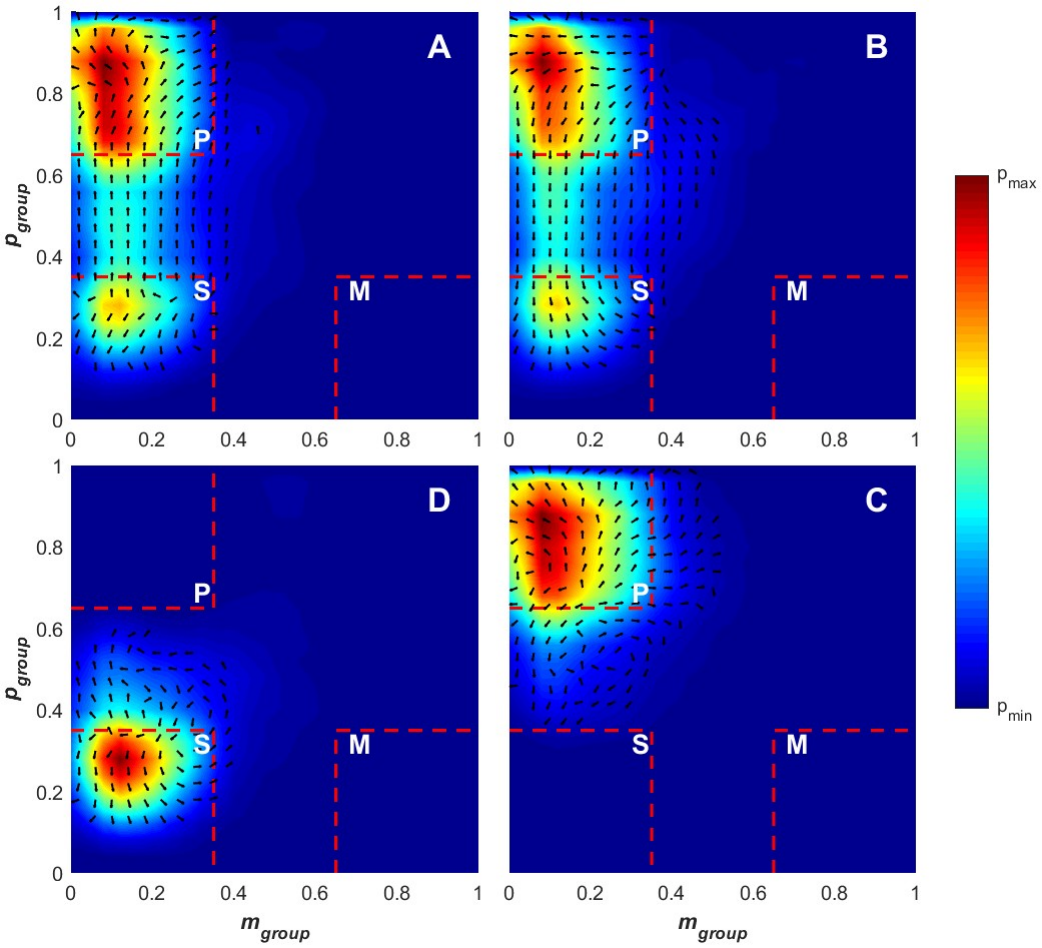
Density plot for paired angular momentum (*m*_*group*_) and polarisation (*p*_*group*_) values across the collective state transitions. (A) Transition from the swarm to polar state, (B) transition from the polar to swarm state, (C) transition from the polar to polar state and (D) transition from the swarm to swarm state. The quivers overlayed on each plot indicate the mean direction of movement in the state-space for time series points that lie within each bin. The divisions of the order parameter space overlayed in red indicate the polar (p), swarm (s), milling (m) and transitional states.

From the results presented in Figs 4A and 7A–7C, it is evident that there are transitions between the collective states, with Fig 7 demonstrating stability within the polar and swarm states during the defensive and attacking game phases respectively. Fig 11 contains density plots and slope fields associated with transitions to and from polar and swarm states (in the *p*_*group*_*—m*_*group*_ plane). Fig 11A and 11B present the transitions from the swarm state to the polar state and polar to swarm state respectively. The density patterns for these transitions are very similar, following a very direct path through the *p*_*group*_*—m*_*group*_ space in both directions. This indicates that the creation of the ordered formation from the unordered swarm and the collapse of the ordered formation into a unordered swarm have similar transitional properties (i.e. in both situations, the group contains similar elements of ordered and unordered movement in *p*_*group*_*—m*_*group*_ space throughout the transitions). This property has been observed in similar studies on schooling fish [18], where transitions in both directions have similar properties across different school sizes. Fig 11C and 11D show the *p*_*group*_*—m*_*group*_ space density for the polar-to-polar and swarm-to-swarm transitions. The density pattern and overlaid quiver plots for the polar-to-polar transition reflect those observed around the polar state of Fig 11A. This indicates that the polar-to-polar transitions more closely reflect those observed in the creation of ordered formations rather than the disintegration into unordered swarms (depicted in 11B). The patterns observed in Fig 11D reflect features seen within the swarm regions in both Fig 11A and 11B.

**Fig 12:**
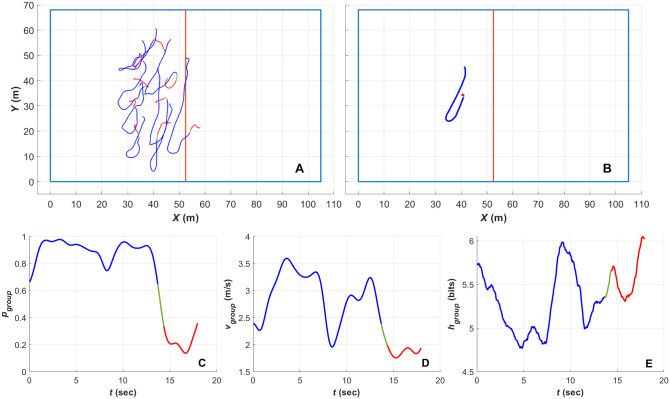
Player position traces and order parameter time series plots for an exemplar polar to swarm transition. The blue, green and red sections of the traces denote the segments of the transition spent in the polar, transitional and swarm states respectively in all plots. (A) The position traces for all players (excluding the goal keeper) across the transition period. (B) The position trace for the centroid (mean position) of the team. (C) The plot of polarisation (*p*_*group*_) time series for the team across the transition period. (D) The plot of the mean group speed (${\overset{-}{\upsilon }}_{group}$) time series for the team. (E) The plot of the of group entropy (*H*_*group*_) time series.

In order to understand the transitions between the ordered and unordered states, Fig 12 provides a series of plots for an exemplar sequence where the team transitions from an ordered polar formation into a swarm. Fig 12A plots the positional traces for all players in the tracked team across the 18 second sequence as they moved on the field (the sequence starts at the blue end of the traces). The blue, green and red sections of the traces indicate the polar, transitional and swarm team collective state segments within the sequence. Fig 12B plots the location of the centroid across the sequence (again with the blue, green sections denoting the polar, transitional and swarm collective states) demonstrating that the team’s formation moved in a ‘hook’ pattern, while maintaining a polar collective state. At the end of the sequence the formation breaks down, with players moving unaligned in different directions as pressure of competitive play is removed. This sequence was selected to demonstrate that ordered, polar, collective movement can be achieved within relatively complex movement patterns. Fig 12C–12E plot the polarisation (*p*_*group*_), mean group speed (${\overset{-}{\upsilon }}_{group}$) and entropy (*H*_*group*_) as function of time across the sequence respectively. Fig 12C demonstrates that the transition from the polar to swarm states occur relatively quickly and follows the pattern demonstrated in Fig 4B, where the group slows down when entering the swarm state (e.g. low polarisation). The effect on the collective motion of the group from the curve present within the hook-shaped movement pattern is evident in Fig 12C–12E with a small perturbation in the group polarisation, a significant dip in the group speed and a spike in the group entropy at approximately 8 seconds. This provides an example of a situation where a polar formation is maintained through a short period of low group speed and high entropy. This demonstrates a characteristic ‘sliding’ formation where the team is maintaining consistent field coverage and individual player spacing while moving to provide pressure on the centre of play (i.e. focused on the position of the ball) in response to the movement of the opposing players.

## Conclusion

This research has presented an exploratory study into the collective states, their transitions and the relationship with the phases of play across a typical game of football. This provides an understanding of where collective order and disorder resides within a typical game and how this links to the tactics for different game phases. The study has demonstrated that the team forms polar and swarm collective states. The team moves between these collective states via direct and relatively fast transitions, even within complex team movements. Higher average group speeds are achieved when the team’s motion is well aligned/polar in nature—an observation that likely arises from the team member’s desire to avoid collisions and maintain a formation. The predictability of the individual player motion is reduced at higher group speeds due to the effect of angular dispersion between individual movements of the players. The collective movement within the defensive game phase is more ordered, compact (i.e. lower surface area) and faster moving compared to the attacking and out-of-play phases. This observation is consistent with conventional tactics, where attacking formations tend to be more spread out to create passing opportunities and breakthroughs. Defensive formations typically attempt to close around the focal point of play to prevent passing opportunities and apply pressure on the attacking player in possession of the ball. This evidence suggests that these defensive tactics may have evolved to provide for faster team movement, allowing the defensive formations to more effectively achieve a numerical superiority around the moving focus of play (e.g. the ball and the closest attacking players). This observation supports previous studies that have suggested training for defensive tactics that resist the disruptions from attacking players that lead to the loss of possession. Conversely, training for offensive tactics that maximise disruption of collective order of the defensive formation may yield better match outcomes. Finally, this work presents a novel view of player movement in team sports, providing further research opportunities focusing on the relationship between collective states and performance within individual segments of play or across whole matches. This is of interest to individual teams for improving match outcomes and the broader community as an approach for characterising the features of exciting and entertaining football matches.
